# GxcC connects Rap and Rac signaling during *Dictyostelium* development

**DOI:** 10.1186/1471-2121-14-6

**Published:** 2013-01-30

**Authors:** Katarzyna Plak, Douwe Veltman, Fabrizia Fusetti, Jetze Beeksma, Francisco Rivero, Peter JM Van Haastert, Arjan Kortholt

**Affiliations:** 1 Department of Cell Biochemistry, University of Groningen, Nijenborgh 7, Groningen, AG, 9747, The Netherlands; 2 Beatson Institute for Cancer Research, Garscube Estate, Switchback Road, Glasgow, G61 1BD, UK; 3 Department of Biochemistry, Netherlands Proteomics Centre, Groningen Biomolecular Sciences and Biotechnology Institute & Zernike Institute for Advanced Materials, University of Groningen, Groningen, AG, 9747, The Netherlands; 4 Centre for Cardiovascular and Metabolic Research, The Hull York Medical School and Department of Biological Sciences, University of Hull, Hull, HU6 7RX, UK

**Keywords:** Rap, Rac, GEF, Development, *Dictyostelium*

## Abstract

**Background:**

Rap proteins belong to the Ras family of small G-proteins. *Dictyostelium* RapA is essential and implicated in processes throughout the life cycle. In early development and chemotaxis competent cells RapA induces pseudopod formation by activating PI3K and it regulates substrate attachment and myosin disassembly via the serine/threonine kinase Phg2. RapA is also important in late development, however so far little is known about the downstream effectors of RapA that play a role in this process.

**Results:**

Here we show that cells expressing constitutively active RapA exhibit a high level of Rac activation. With a pull-down screen coupled to mass spectrometry, we identified the Rac specific guanine nucleotide exchange factor, GxcC, as Rap binding partner. GxcC binds directly and specifically to active RapA and binds to a subset of *Dictyostelium* Rac proteins. Deletion studies revealed that this pathway is involved in regulating *Dictyostelium* development.

**Conclusions:**

GxcC provides a novel link between Rap and Rac signalling and is one of the Rap effectors regulating the progression of multicellular development.

## Background

Small G-proteins are molecular switches that cycle between an inactive GDP- and active GTP-bound state. Activation is dependent on helper proteins that facilitate release and exchange of bound nucleotide: GEFs (Guanine nucleotide Exchange Factors), while its deactivation is regulated by GAPs (GTPase activating proteins) that stimulate the naturally low GTPase activity [1]. Only in the active GTP bound form, the switch region of small G-proteins can interact with Ras-associating (RA) or Ras binding domains (RBD) of effector proteins [2,3]. In this way Small G-proteins provide tight control over the cell response to extracellular stimuli by translating the signaling cascades inside the cell [4].

In *Dictyostelium discoideum* small G-proteins are essential for a wide variety of processes, including regulation of the cytoskeleton, chemotaxis, cell division and multicellular development [5,6]. Due to its genetic tractability and high conservation of many important signaling pathways *Dictyostelium* has proven to be an excellent model for studying small G-protein signaling. During the vegative state, *Dictyostelium* are single-celled amoeba that feed on bacteria. Upon starvation, cells undergo a tightly regulated developmental process in which they secrete and chemotax toward cAMP, resulting in multicellular fruiting bodies [7,8].

Rap proteins belong to the Ras superfamily of small G-proteins. In mammalian cells Rap is important for cellular adhesion, differentiation and cell proliferation [9,10]. *Dictyostelium* RapA is essential and is implicated in processes throughout the life cycle. In vegetative and chemotaxis competent cells RapA induces pseudopod formation by activating PI3K [11], and it regulates substrate attachment and myosin II disassembly via the serine/threonine kinase Phg2 [12,13]. Strains with a deletion of *rapGAP2* or *rapGAP3* showed defects in cell patterning and morphogenesis [14,15], indicating that RapA is also important in late development. However so far nothing is known about downstream effectors of RapA that play a role in the progression of multicellular development.

Here we show that activation of RapA results in increased level of activated Rac proteins. To gain further insight into the link between these pathways, we have used a proteomic approach. Recombinant RapA was used as bait in pull-down screens and interacting proteins were identified by mass-spectroscopy. One of the binding partners, GxcC provides a potential link between Rap and Rac activation and deletion studies reveal that this pathway is involved in regulating development of *Dictyostelium* cells.

## Results and discussion

### Rap regulates Rac activation

**Figure 1:**
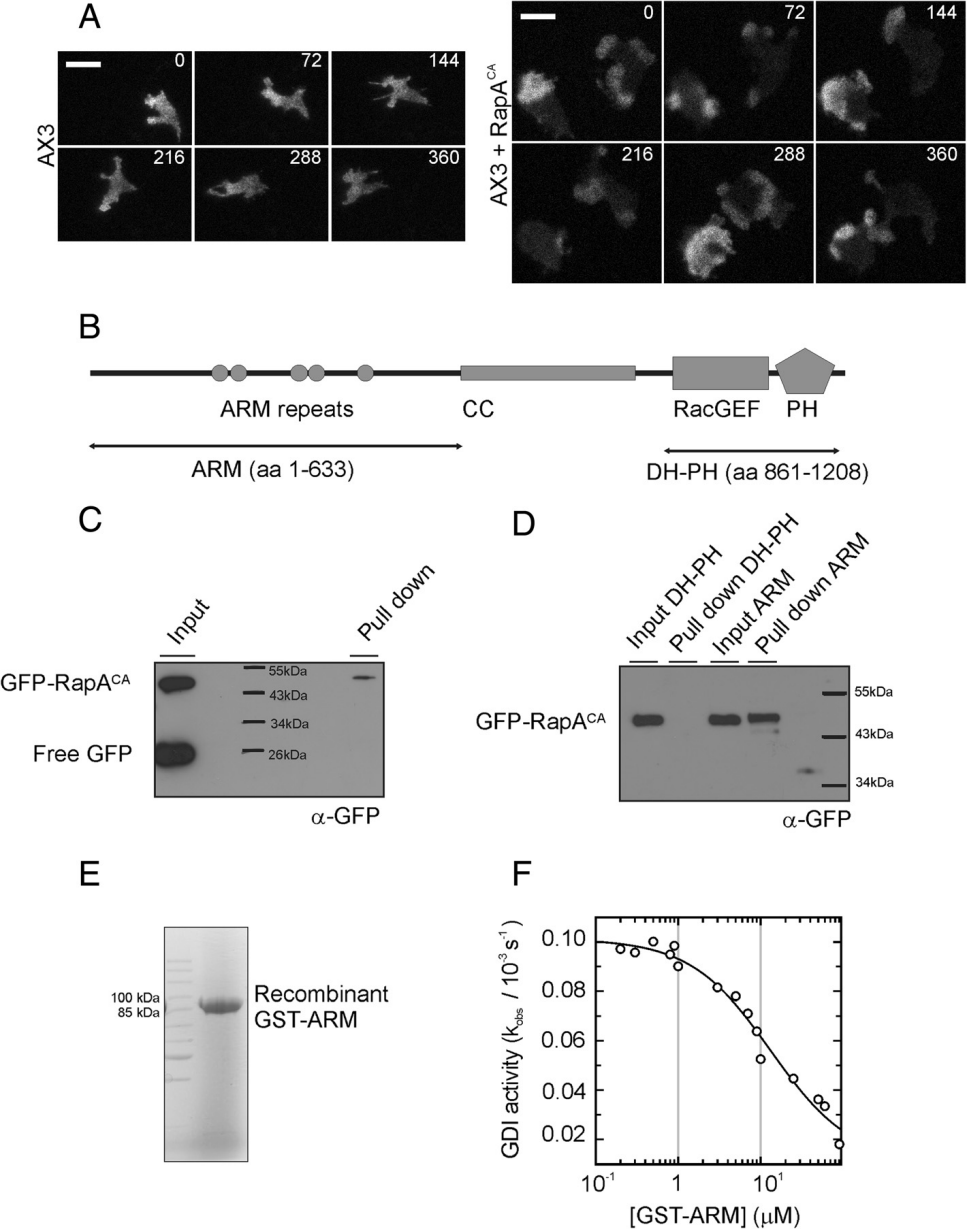
**GxcC is a specific RapA interaction partner.** (**A**) Dynamics of Rac activation visualized with CRIB-GFP. Cells were developed in shaken suspension for 3 hours and pulsed with 50 nM cAMP for another 2 hours. Developed cells were transferred to a glass bottom dish and overlayed with PB + 0.4% agarose. Images were collected using TIRF microscopy (original experiments are shown in Additional file 1: Movie 1 and Additional file 2: Movie 2). Scale bar is 10 μM. (**B**) Domain composition of GxcC. ARM – Armadillo repeats, CC – Coiled Coil region, RacGEF – Guanine Nucleotide Exchange factor for Rac, PH – Pleckstrin Homology domain. Arrows indicate the boundaries of truncated constructs. (**C**) Pull down in *Dictyostelium* lysate with GST-GxcC as bait and GFP-RapA^CA^ as prey. (**D**) Pull down with GST-ARM or GST-DH-PH as bait and GFP-RapA^CA^ as prey. The amount of prey was detected by western blotting using antibody specific for GFP (**E**) SDS PAGE analysis of recombinant GST-ARM (95 kDa) purified from *E coli*. (**F**) GDI assay in which the dissociation rate of mGppNHP from RapA was measured in the presence of varying concentrations of GST-ARM. The addition of increasing concentrations of the effector results in a concentration-dependent decrease of the observed rate constant (k_obs_).

Rap proteins are involved in the regulation of the actin cytoskeleton. In mammals Rap promotes cell spreading via the RacGEFs Vav2 and Tiam1 [16]. In *Dictyostelium* expression of constitutive active RapA (RapA^CA^) results in an increased actin response and F-actin polymerization is induced at the sites were RapA is activated in response to cAMP [17]. However the pathways and mechanism by which Rap regulates cytoskeleton reorganization are not completely understood. The dynamics of Rac activation in *Dictyostelium* cells can be monitored using the fluorescent probe CRIB-GFP that consists of the CRIB domain of *Dictyostelium* PakB, that specifically binds to the active form of Rac [18,19]. To test if RapA can indeed activate the Rac pathway we coexpressed RapA^CA^ and CRIB-GFP and visualised active Rac using total internal reflection microscopy. In random moving wild type (AX3) cells there is a broad patch of active Rac in pseudopods. In unstimulated developed cells, there is usually a single (1.3 ±0.5, n = 20) stable patch that slowly migrates along with the pseudopod (Figure 1A, Additional file 1: Movie 1). Cells expressing RapA^CA^ are flattened and unpolarized in appearance [13]. In these cells, active Rac patches are highly motile (Figure 1A, Additional file 2: Movie 2). Patches grow to unusually large size and often break up into multiple (2.4 ± 0.9, n = 20) patches, which leads to multiple simultaneous protrusions.

### GxcC is a specific RapA effector

To identify Rap effectors we performed a pull-down screen in *Dictyostelium* lysate with purified RapA loaded with GppNHp as bait. By using mass-spectrometry a putative Rac GEF (GxcC - **G**uanine e**X**change factor for ra**C**, UniProt: Q54P24) was identified as potential binding partner. GxcC has N-terminal ARM repeats, followed by a coiled-coil region, a predicted RhoGEF (DH) domain and a Pleckstrin Homology (PH) domain (Figure 1B). *Dictyostelium* cells do not have Rho or Cdc42 homologues, but RhoGEF domain containing proteins are thought to regulate the function of the *Dictyostelium* Rac family [20].

To confirm that RapA can interact with GxcC, we co-expressed GST-fused GxcC and GFP-tagged RapA^CA^ in *Dictyostelium* and performed pull-down experiments using GSH beads. Using GST-GxcC as a prey we were able to pull-down GFP-RapA^CA^, whereas free GFP that is present in the same sample does not bind to GxcC (Figure 1C).

Mammalian Rap1 binds to the DH-PH tandem of the RacGEFs, TIAM and VAV2 [17,21]. To determine which domain of GxcC is responsible for RapA binding, we expressed truncated GxcC constructs (Figure 1B) and performed pull down experiments. Figure 1D shows that the DH-PH tandem is not binding to active RapA and that binding between RapA and GxcC is mediated by the ARM domain containing N-terminal fragment. This interaction is not disrupted by the actin inhibitor LantrunculinA, indicating that RapA binding to GxcC does not need an intact cytoskeleton (Additional file 3: Figure S1A). To determine the specificity of the RapA/GxcC interaction we tested the ability of RasC and RasG to bind to GxcC. GxcC does not bind to active RasG and only very weakly to active RasC (Additional file 3: Figure S1B).

**Figure 2:**
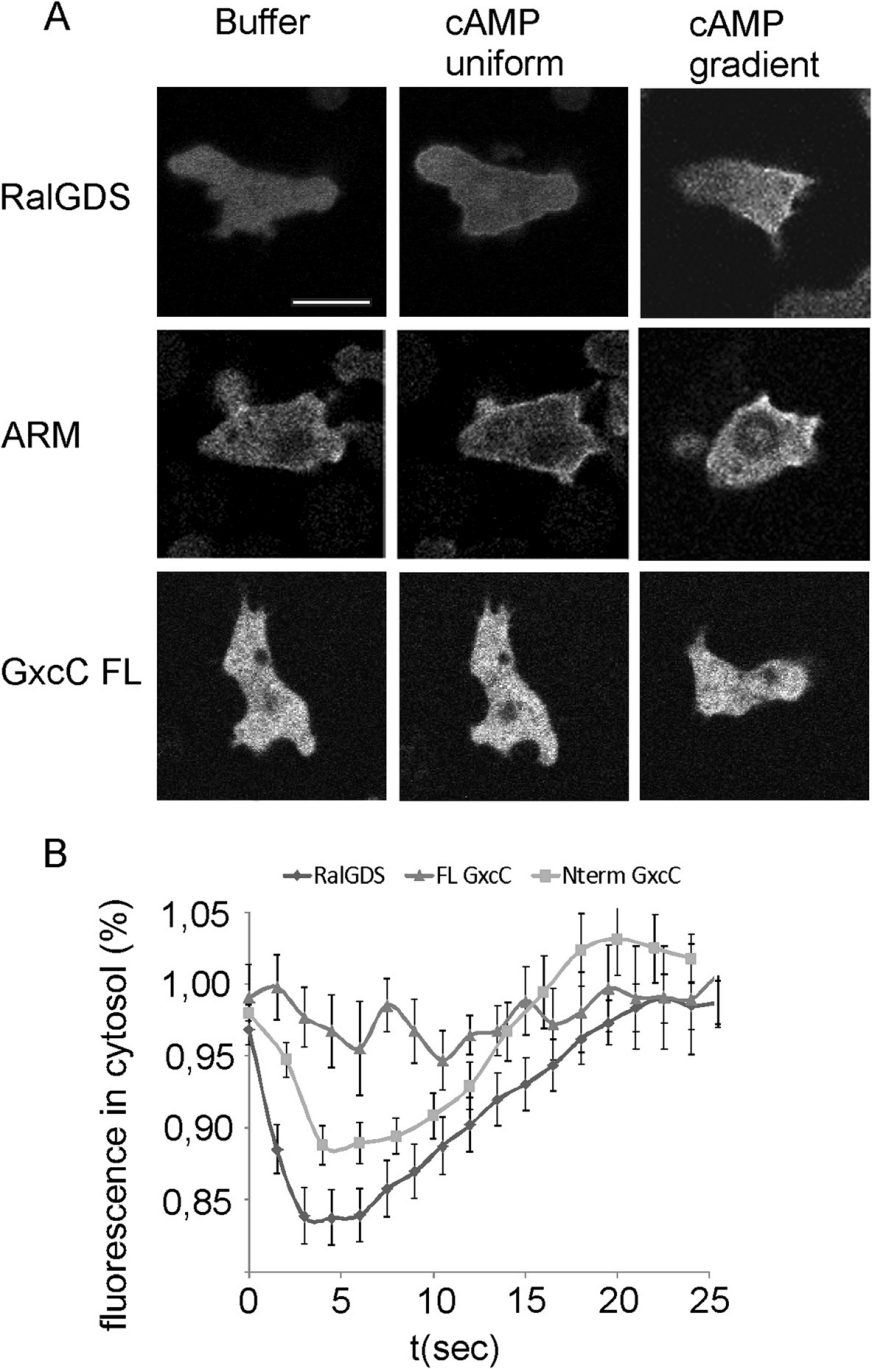
**Localization and translocation dynamics of GxcC ARM coincides with Rap activation.** (**A**) Confocal images of starved cells expressing GFP-RalGDS, GFP-ARM or GFP-GxcC in buffer, 4 sec after cAMP stimulation or in a gradient of cAMP (**B**) Time-course of RalGDS, GFP-ARM or GFP-FL GxcC translocation from cytoplasm to the membrane upon stimulation with 1μM cAMP. Shown are the mean and SEM of at least 9 cells.

Rap activation *in vivo* can be monitored with the previously published GFP-RalGDS reporter, which specifically binds to Rap-GTP [22]. Upon uniform stimulation with cAMP, GFP-RalGDS transiently translocates uniformly to the cell boundary, maximum peaking after 3–6 sec after stimulation, followed by a return to the cytoplasm around 20 sec (Figure 2A + B) [13]. N-terminal GxcC shows a highly similar response; it is recruited from the cytoplasm to the cell boundary upon cAMP stimulation, with a maximum drop in the cytoplasm after 5 sec after stimulation and return to basal after 20 sec (Figure 2A + B). Upon application of a chemoatractant gradient to wild-type cells, both GFP-RalGDS and N-terminal GxcC localize at the side of the cell facing the gradient (Figure 2A). On the contrary, full length GxcC is uniformly distributed in the cytosol and doesn’t alter localization upon stimulation with cAMP (Figure 2A + B), suggesting GxcC is in an auto-inhibited state and may need additional specific inputs for translocation to the membrane and/or RapA is locally activating GxcC rather than regulating its localization. These data show that the localization and kinetics of RapA activation coincides with the localization of N-terminal GxcC, indicating an interaction *in vivo*.

To address if the interaction between GxcC and RapA is direct and nucleotide dependent, the ARM domain of GxcC (Figure 1E) and RapA were expressed and purified from *E*. *coli* and subsequently used in a Guanine nucleotide dissociation inhibition (GDI) assay. In this experiment RapA loaded with mGppNHp, a hydrolysis-resistant fluorescent GTP analogue, is incubated with an excess of unlabelled nucleotide. The resulting nucleotide exchange can be monitored as decay in fluorescence, from which the rate constant k_obs_ is calculated. The interaction of an effector with the GTP-bound G-protein stabilizes the interaction between the G-protein and the nucleotide and this stabilization results in decreased dissociation of the nucleotide from the G-protein/ nucleotide/ effector complex [23]. The addition of increasing concentrations of the purified GxcC ARM domain resulted in a concentration dependent decrease of k_obs_. Using this dependency we determined an affinity (K_d_) of 13.3 μM (Figure 1F) for the interaction of RapA and GxcC. Although this affinity seems to be rather low, it is similar to the ones described for the interaction between RapA and Phg2 [12], RapA and PI3K [11] and human Rap with several effectors [24]. Measurements with RapA bound to mGDP or mGppNHp loaded RasC and RasG, didn’t show any GDI effect in the presence of GxcC, indicating the interaction is nucleotide dependent and specific.

All together our results show that active RapA specifically binds to the ARM repeat region of GxcC *in vitro* and *vivo*.

#### GxcC binds to Rac proteins

**Figure 3:**
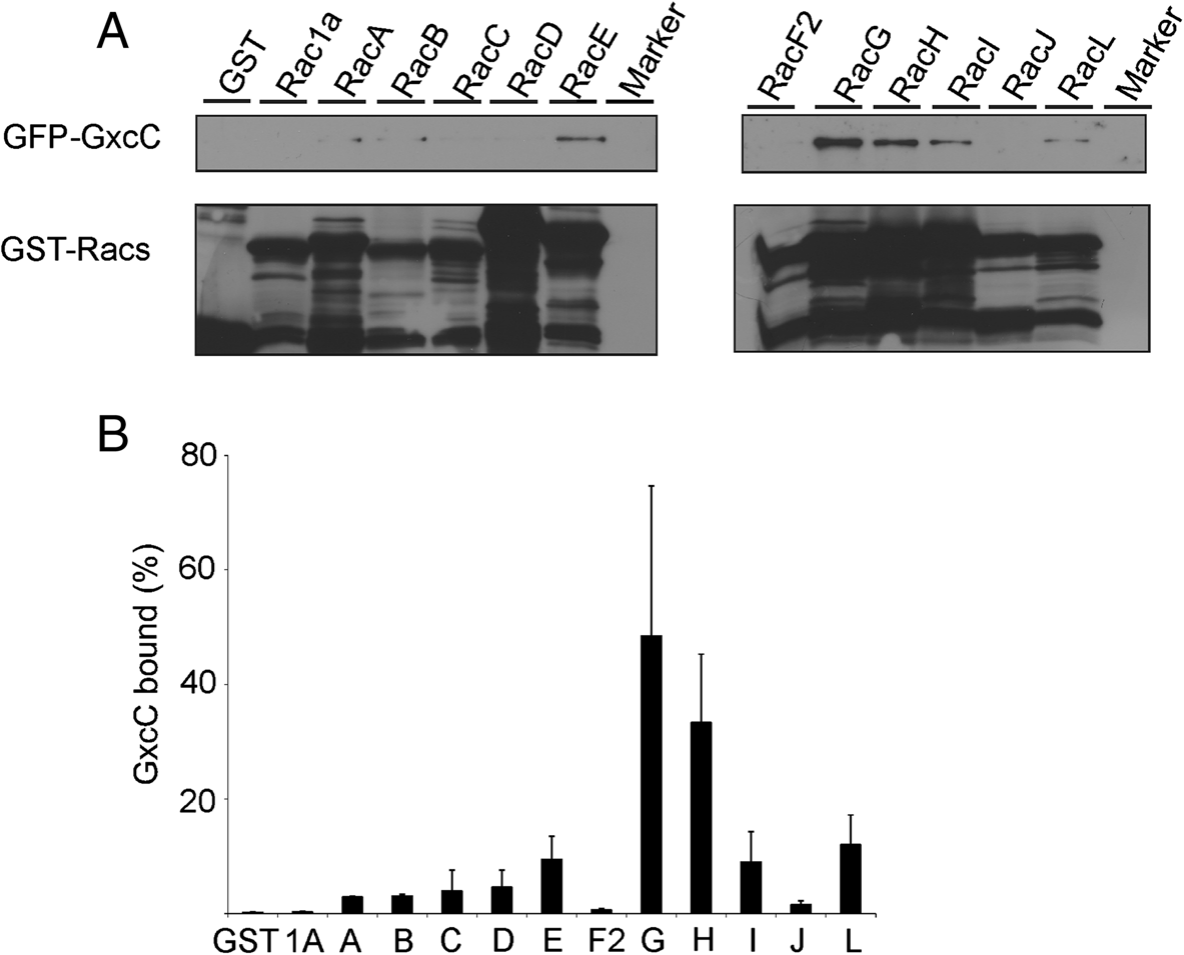
**GxcC binds to a subset of *Dictyostelium *Rac proteins.** (**A**) GST and various purified GST-Rac proteins were incubated with GFP-GxcC cell lysate. Beads were precipitated and the amount of GFP-GxcC and bait was detected by western blotting using antibody specific for GFP or GST, respectively (**B**) The amount of detected proteins was quantified using ImageJ and normalized to the amount of GxcC protein used in the assay. Shown is the percentage of bound GxcC as mean and SEM of three independent experiments.

The *Dictyostelium* genome encodes for 18 Rac proteins, whereas Rho and CDC42 are absent [20]. To determine the downstream targets of GxcC, binding to *Dictyostelium* Rac proteins was determined. The Rac proteins were expressed and purified from *E*. *coli*, bound to GSH beads and subsequently incubated with lystate of vegetative GFP-GxcC *Dictyostelium* cells in the presence of EDTA. Imunnoblotting was used to detect bait (GST-Rac) and prey (GFP-GxcC) protein. GxcC showed the highest affinity to RacG, RacH, RacE, RacI and RacL whereas hardly binding to Rac1A, RacF2 and RacC could be detected (Figure 3), indicating specificity of GxcC binding in the pull-down. To get complete insight in the downstream targets of GxcC we tried to perform *in vitro* nucleotide exchange assays, but unfortunately we were not able to isolate a stable recombinant GxcC DH-PH fragment. We were able to isolate small quantities of full length GxcC from *Dictyostelium* cells, but this protein didn’t show exchange activity on any of the tested Rac proteins. This lack of activity could be due to the quality of the isolated protein, or consistent with the translocation data may suggest that full length GxcC is in an auto-inhibited state (unpublished results).

Only a few of the Rac proteins have been characterized in detail. RacG which shows the highest binding is involved in cAMP chemotaxis, filopodia formation and phagocytosis [25]. RacH which also shows high binding to GxcC has a role in vesicular trafficking [26], RacE is known to play a role in orchestrating cell division [27], and RacL is a yet uncharacterized Rac protein that is expressed at late stages of *Dictyostelium* development [28].

Together the data shows that GxcC can bind specific Rac proteins. The presence of the predicted RhoGEF DH-PH domain suggest that GxcC most likely is able to activate these interacting proteins, it is however also unlikely that it is the only exchange factor for these Rac’s.

### GxcC plays a role during development

To characterize the function of GxcC *in vivo* we generated cells lacking *gxcC* by homologous recombination. Three independent knock out clones have been isolated and the integration in the proper loci was confirmed by PCR (Additional file 3: Figure S2). A rescue cell line was prepared by overexpressing a full length GxcC construct in the knock out background. The role of GxcC in chemotaxis was tested using a micropipette assay. In a gradient of cAMP, wild type cells were very polarized and robustly moved towards the pipette with a chemotaxis index of 0.79 ± 0.09 and speed of 9.57 ± 2.97 μm/min. Cells lacking *gxcC* migrate with similar efficiency (chemotaxis index of 0.81 ± 0.07), and move slightly faster (speed of 15.2 ± 3.34 μm/min), as wild-type cells. The knock out cells did not show defects in cytokinesis, also the cell attachment and cell morphology of *gxcC*-null and GxcC rescue cells is similar to that of wild-type, suggesting that GxcC does not play an important regulatory role in these processes. Consistently, *gxcC*- null cells expressing RapA^CA^ still possessed the characteristic Rap^OE^ phenotype; cells were flat, highly adhesive extended many substrate attached pseudopodia with highly mobile Rac patches (unpublished results).

**Figure 4:**
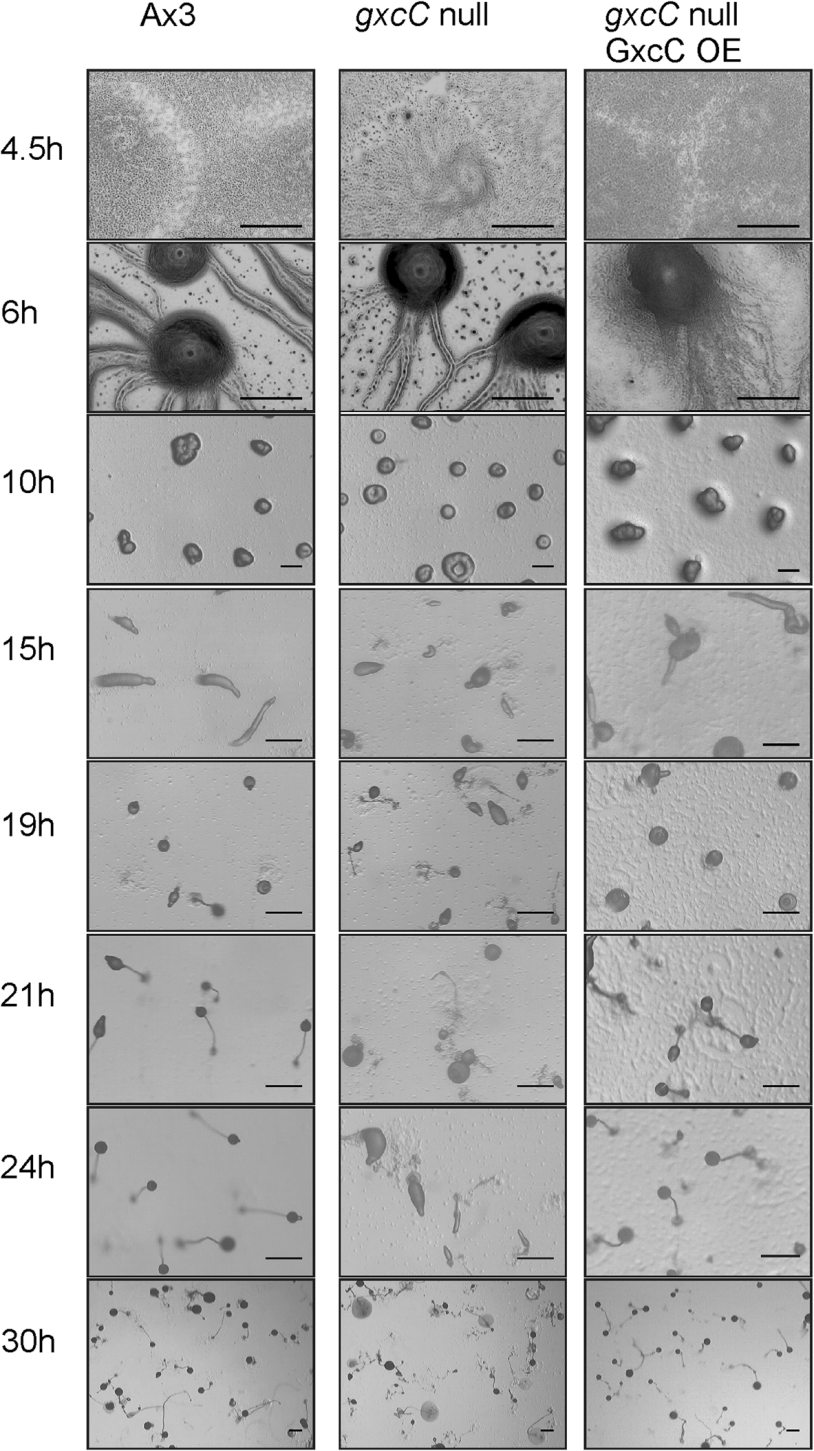
***gxcC*-null cells have a defect in multicellular development.** Cells were plated on non-nutrient agar and allowed to develop. Pictures were taken at the indicated time points after starvation. Scale bar represents 0.5 mm.

Because Rap activation is required for proper cell sorting and fruiting body formation [14,15], we examined the phenotype of *gxcC*-null during *Dictyostelium* development. In wild-type (AX3), aggregation centers were formed after 6 hours, slugs were formed after 15 hours, Mexican hats were visible after 18 hours and after 24 hours cells culminates into fruiting bodies (Figure 4). Cells lacking *gxcC* formed territories and finished streaming phase at the same time as wild-type parent. However, they were severely delayed in forming slugs and Mexican hat structures, and eventually formed fruiting bodies after 30 hours of starvation. This developmental defect could be completely reverted by re-expressing N-terminal GFP tagged GxcC (Figure 4).

Together the data shows that GxcC plays a role in the regulation of *Dictyostelium* development.

## Conclusions

With a mass spectrometry based screen we here identified the Rac guanine nucleotide exchange factor, GxcC as Rap binding partner. Our biochemical data shows that the interaction between GxcC and RapA is both specific and direct, suggesting a potential link between Rap and Rac signalling. In mammalian cells Rap promotes cell spreading by localization of the RacGEFs Vav2 and Tiam [16]. *Dictyostelium* Rap is a key regulator of adhesion, chemotaxis and development [11,13,15]. GxcC only regulates *Dictyostelium* late development and is not required for adhesion or chemotaxis. Starved cells expressing Rap^CA^ have increased Rac activation, suggesting there has to be additional modes of Rap regulation of the Rac pathway. A recent paper by Jeon et al., suggests that *Dictyostelium* RacGEF1 might be one of these connection between Rap and Rac signalling [17]. To get more insight in these pathways it will be important to identify further regulators and downstream effectors of small G-protein signaling; our mass-pull-down screen could be important in this enterprise.

## Methods

### Cell culture and preparation

AX3 *Dictyostelium* cells were used as wild-type strain. Cells were grown in HL5-C media (Formedium) either on nunclon-coated petri dishes or in shaking flasks. The indicated GxcC overexpressing and knock out constructs were transformed to *Dictyostelium* cells. For selection, the media was supplemented with the appropriate antibiotics; 10 μg/ml Blasticidine S for KO cell lines, and 10 μg/ml Genticin or 50 μg/ml Hygromycine B for overexpression cell lines. To induce starvation cells were washed with PB buffer (10 mM KH_2_PO_4_/Na_2_HPO_4_, pH 6.5) and plated out on layers of non-nutrient agar (1,5% agar in PB).

### Construction of plasmids

The *gxcC* gene was amplified from cDNA by PCR using the primers sequences: 5^′^-GGGATCCATGCCAATTCAATTTGATGC-3^′^ and 5^′^-GCTAGCTTATTTTTTTGAAGTTAAAACTTTTG-3^′^. An N-terminal construct of GxcC (base pairs 1–1911) was amplified using the forward primer 5^′^-GGATCCATGCCAATTCAATTTGATGC-3^′^ and reverse primer 5^′^-GCTAGCTTCAGCTTCATCTTCATCATCTTC-3^′^). A C-terminal GxcC (basepairs 2578–3648) construct was obtained with the primers: 5^′^-GGATCCGATCAACAAGATAAAGCTTCAC3^′^ and 5^′^GCTAGCTTATTTTTTTGAAGTTAAAACTTTTG-3^′^). The obtained PCR fragments were digested with BamHI and ligated into the BglII site of the previously characterized *Dictyostelium* extrachromosomal plasmids pDM314 (N-terminal GST) and pDM317 (N-terminal GFP) [29]. For expression in *E*. *coli* the digested N-terminal GxcC fragment was ligated into the BamHI site of pGEX 4T3 (GE Healthcare, N-terminal GST).

The *gxcC* gene disruption construct was created by amplifying the GxcC fragment from position 1660 to 3648 (5^′^-GGATCCAGAAATCGTAAGGTTATGAATG-3^′^, 5^′^-GCTAGCTTATTTTTTTGAAGTTAAAACTTTTG-3^′^). This fragment was ligated in a pBluescript (Stratagene) vector and a BSR cassette was inserted in the Eco72I site of the resulting vector. The KO fragment was linearized by restriction digestion and isolated.

### RapA pull down screen

GST-RapA fusion protein was purified as described before [12]. The purified protein was preloaded with non-hydrolyzable GTP analogue GppNHp and 3–5 mg of protein was prebound to a GSH-column. Lysate of *Dictyostelium discoideum* vegetative cells was circulated over the column overnight to allow binding of possible interaction partners to the activated GST-RapA protein. Unbound proteins were washed away from the column and RapA and its interaction partners were subsequently eluted with glutathione elution buffer.

### Protein identification by mass spectrometry

Protein samples were concentrated and separated by 1D-SDS-PAGE. After Coomassie staining each lane was cut into 24 and subjected to in-gel digestion with trypsin (Trypsin Gold, Promega), prior reduction with 10 mM DTT and alkylation with 55 mM iodoacetamide. Peptide mixtures were trapped on C18 reversed phase EASY-Column and separated on a 100 mm C18 reversed phase column (75 μm × 100 mm, 3-μm particle size, Thermo Scientific) using a linear gradient from 0% to 35% B (A = 0.1% formic acid; B = 100% (v/v) acetonitrile, 0.1% formic acid) over 70 min at a constant flow rate of 300 nL/min. Nanoflow LC-MS/MS was performed on an EASYII LC system (Thermo Scientific) coupled to an LTQ-Orbitrap XL mass spectrometer (Thermo Scientific) operating in positive mode. MS scans were acquired in the Orbitrap in the range from 350 to 1800 m/z, with a resolution of 60,000 (FWHM). The 7 most intense ions per scan were submitted to MS/MS fragmentation (35% Normalized Collision Energy™) and detected in the linear ion trap. Peak lists were obtained from raw data files using the Proteome Discoverer v 1.3 software. Mascot (version 2.1, MatrixScience) was used for searching against a sequence database obtained by combining the *E*. *coli* with the *Dictyostelium* proteome sequences. The peptide tolerance was set to 40 ppm and the fragment ion tolerance to 2.0 Da, using semi-trypsin as protease specificity and allowing for up to 2 missed cleavages. Oxidation of methionine residues, deamidation of asparagine and glutamine, and carboamidomethylation of cysteines were specified as variable modifications. Peptide and protein identifications were further validated with the program Scaffold (Version Scaffold_3.2, Proteome Software Inc., Portland, OR). Protein identifications based on at least 2 unique peptides identified by MS/MS, each with a confidence of identification probability higher than 95%, were accepted.

### Protein purification and GDI assays

NtermGxcC (aa 1–633) was expressed from a pGEX4T3 plasmid containing a N-terminal GST side. Recombinant protein was purified by GSH affinity and size exclusion chromatography. The purified protein was used in a GDI assay as previously described [23].

### GxcC pull down experiments

*Dictyostelium* cells expressing GST tagged bait proteins and GFP tagged prey proteins were collected and lysed in 2ml of LB buffer (10 mM Na_2_HPO_4_ pH 7.2, 1% Triton X-100, 10% glycerol, 150mM NaCl, 10 mM MgCl_2_, 1 mM EDTA, 1 mM Na_3_VO_4_, 5 mM NaF and protease inhibitor cocktail (Roche). Precleared cell lysate was mixed with GSH beads (GE Healthcare) and incubated with rotation overnight at 4°C. Unbound proteins were washed away with PBS and prey proteins were detected by Western Blot with α-GFP primary antibody (SC9996).

### Microscopy

Confocal images were taken with a Zeiss LSM 510 META-NLO confocal laser scanning microscope with a Zeiss plan-apochromatic × 63 numerical aperture 1.4 objective. Femtotip micropipette filled with 0.1 mM cAMP was introduced to the field of cells to induce chemotaxis towards increasing cAMP gradient. The response to uniform stimulation with cAMP was tested by introducing a broken micropipette filled with 1 μM cAMP and quantified using ImageJ software [30]. TIRF microscopy was performed on a Nikon Eclipse TE2000-U that was fitted with a custom TIRF condenser and a Nikon 1.45 NA 100x Plan Apo TIRF objective and an Evolve 512 EMCCD camera.

### Rac binding assays

GST-Rac proteins were expressed and coupled to GSH beads as previously described [20]. Pre-cleared lysate of vegetative *Dictyostelium* cells overexpressing GFP-GxcC was prepared as described above and incubated with the GSH beads coupled GST-Rac proteins. Binding was allowed at 4°C for 1 hour, unbound proteins were washed away, and bait and prey proteins were detected by Western Blot with α-GFP antibody (SC9996) and α-GST antibody (GE-Healthcare). Image J software was used to quantify binding specificity [30].

## Authors’ contributions

KP, PJMvH, and AK designed the experiments and wrote the paper. KP performed most biochemical and cellular experiments. JB carried out initial biochemical and localization experiments, DV did the *in vivo* Rac activation experiments and FF the MS analysis. FR provided constructs and helped with the GST-Rac pull-downs. All authors read and approved the final manuscript.

## Authors’ information

The authors declare that they have no competing interest. Supplementary Information is linked to the online version of the paper. Correspondence and request for materials should be addressed to A.Kortholt@rug.nl.

## Supplementary Material

Additional file 1: Movie 1 Visualisation of active Rac in Wild Type cells. AX3 cells expressing the active Rac marker CRIB-GFP (vector pDM841) were induced to develop by starving them for 4 hours and pulsing them for 2 hours with 100 μM cAMP pulses every 6 minutes. Cells were transferred to a glass-bottom dish and overlayed with a thin layer of 0.4% agarose in development buffer. Fluorescence images (green) were collected using TIRF illumination at 1 frame per 2 seconds. The brightfield image is shown in dark blue. The field of view is 83x83 micrometers. Click here for file

Additional file 2: Movie 2 Visualisation of active Rac in RapG12V overexpressing cells. AX3 cells expressing the active Rac marker CRIB-GFP (vector pDM841) and constitutive active Rap (G12V mutation) under a folate-responsive promoter were induced for 24 hours with 1mM folate. Cells were induced to develop by starving them for 4 hours and pulsing them for 2 hours with 100 μM cAMP pulses every 6 minutes. Cells were transferred to a glass-bottom dish and overlayed with a thin layer of 0.4% agarose in development buffer. Click here for file

Additional file 3: Figure S1 GxcC is a specific RapA interaction partner. (A) Binding between GxcC and active RapA is independent of filamentous actin. Pull down with GST-RapA^CA^ as bait and GF-PARM as prey. *Dictyostelium* cells overexpressing bait and prey proteins were incubated in PB or PB with 5 μM Latrunculin A (Sigma) for 10 minutes prior to lysis. The amount of prey was detected by western blotting using antibody specific for GFP. (B) GxcC binds specifically to RapA. Pull down with GST-RapA^CA^, GST-RasC^CA^ GST-RasG^CA^ or GST alone as bait and GF-PARM as prey. Western Blot with GFP antibody was used to detect bound ARM. **Figure S2**. Construction of *gxcC* KO cell line. (A) Plasmid map of the *gxcC* KO construct. BSR cassette was inserted at position 2630. Indicated primers were subsequently used for testing in locus integration. (B) *gxcC* KO construct was integrates in locus. Three independent clones show 2165bp high band corresponding to disrupted *gxcC* gene. Control sample of AX3 wt cells DNA shows a band of 1077bp, which indicates undisrupted *gxcC*. (PDF 30 kb) Click here for file
