# Supplementary figures and images for: GxcC connects Rap and Rac signaling during Dictyostelium development
Source: BMC Cell Biol. 2013 Jan 30;14:6. doi: 10.1186/1471-2121-14-6 (PMC3675359; doi:10.1186/1471-2121-14-6)

FigS1

A

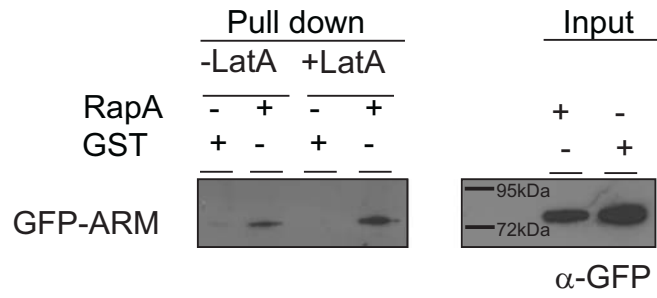

B

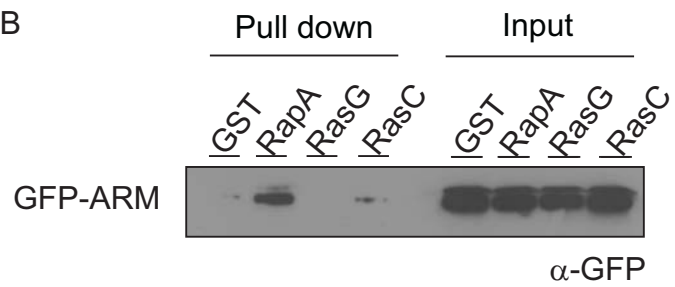

FigS2

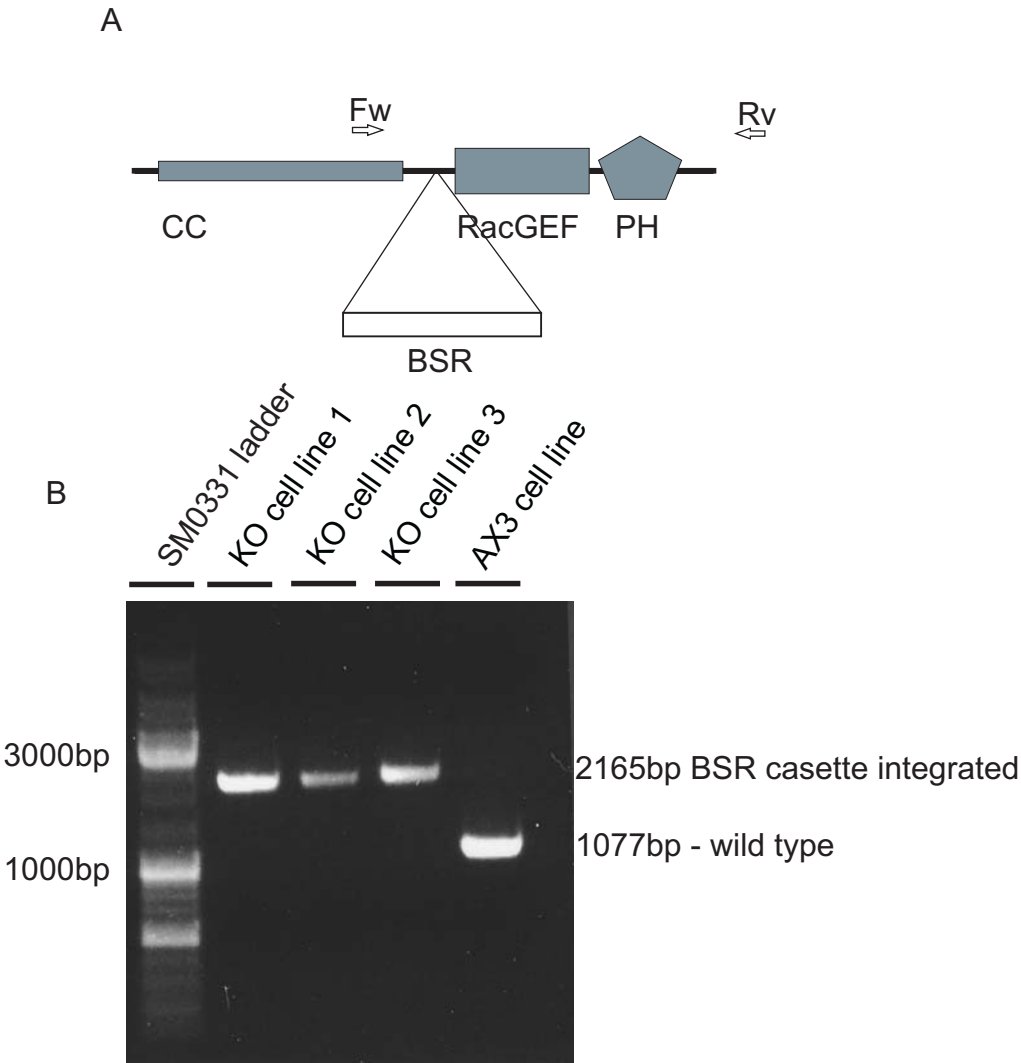

Supplement: Additional file 3: Figure S1 — GxcC is a specific RapA interaction partner. (A) Binding between GxcC and active RapA is independent of filamentous actin. Pull down with GST-RapACA as bait and GF-PARM as prey. Dictyostelium cells overexpressing bait and prey proteins were incubated in PB or PB with 5 μM Latrunculin A (Sigma) for 10 minutes prior to lysis. The amount of prey was detected by western blotting using antibody specific for GFP. (B) GxcC binds specifically to RapA. Pull down with GST-RapACA, GST-RasCCA GST-RasGCA or GST alone as bait and GF-PARM as prey. Western Blot with GFP antibody was used to detect bound ARM. Figure S2. Construction of gxcC KO cell line. (A) Plasmid map of the gxcC KO construct. BSR cassette was inserted at position 2630. Indicated primers were subsequently used for testing in locus integration. (B) gxcC KO construct was integrates in locus. Three independent clones show 2165bp high band corresponding to disrupted gxcC gene. Control sample of AX3 wt cells DNA shows a band of 1077bp, which indicates undisrupted gxcC. (PDF 30 kb) [file 1471-2121-14-6-S3.pdf]
